# Supplementary material for: A Likelihood Approach for Real-Time Calibration of Stochastic Compartmental Epidemic Models
Source: PLoS Comput Biol. 2017 Jan 17;13(1):e1005257. doi: 10.1371/journal.pcbi.1005257 (PMC5240920; doi:10.1371/journal.pcbi.1005257)
Supplement: S1 File — (TAR.GZ) [file pcbi.1005257.s014.tar.gz › HSPH_Online-SI-Revision/Additions/EnKF08/n100-extreme-SEITR/n100-extreme-SEITR_EnKF_table-4.pdf]

| | | | | | | | | | | | | | | | | | | | | | | | | | | | |
|---|---|---|---|---|---|---|---|---|---|---|---|---|---|---|---|---|---|---|---|---|---|---|---|---|---|---|---|
| 1 | R0 | {1.38654, 9.85565} | {2.24482, 5.46643} | {{0.903694, 0.689491}, {3., 19.4947}} | {84.8287, 83.6666} | inf | 4942. | 6528.53 | {526.564, 6065.41} | 59.4 | 0.364945 | newly | 7106. | 10 253.4 | {541., 11 060.} | 110.335 | 0.473712 | Reff | 1.09645 | 0.436618 | {0.154948, 0.644891} | 64.5376 | ar | 92 379. | 185 453. | {165 203., 228 186.} | 100.752 |
| 2 | R0 | {2.36629, 12.2029} | {2.15572, 4.08901} | {{1.14832, 2.65527}, {3., 4.99802}} | {35.3526, 66.4915} | inf | 1894. | 16 978.6 | {5569.51, 57 532.2} | 512.831 | 0.61759 | newly | 3034. | 18 159.2 | {6351., 56 814.} | 500.05 | 0.596449 | Reff | 2.22479 | 0.35329 | {0.0882591, 0.784287} | 84.1507 | ar | 99 022. | 155 442. | {75 222., 261 465.} | 65.8601 |
| 3 | R0 | {2.32253, 12.836} | {2.53421, 7.1759} | {{2.16604, 4.13176}, {3., 13.4516}} | {20.1947, 47.366} | inf | 1753. | 25 879.5 | {4794.6, 57 065.4} | 883.019 | 0.839459 | newly | 2684. | 27 073.7 | {4575., 72 658.} | 909.525 | 0.827224 | Reff | 2.19238 | 0.998064 | {0.380008, 2.11116} | 54.7875 | ar | 98 997. | 156 039. | {101 025., 235 687.} | 62.7987 |
| 4 | R0 | {1.79075, 11.2058} | {2.06167, 4.0232} | {{0.985519, 2.64795}, {3., 4.93211}} | {48.085, 64.0972} | inf | 2803. | 14 437. | {3227.32, 34 749.1} | 265.852 | 0.460299 | newly | 3933. | 16 349.1 | {5109., 44 974.} | 324.99 | 0.501186 | Reff | 1.61667 | 0.385342 | {0.0514258, 0.789394} | 76.3806 | ar | 96 920. | 155 169. | {72 959., 202 061.} | 70.0871 |
| 5 | R0 | {2.26952, 9.62371} | {2.56865, 7.03781} | {{2.16374, 4.17057}, {3., 14.7178}} | {19.9452, 40.406} | inf | 1270. | 22 320.1 | {3720.49, 57 051.7} | 1035.83 | 0.910467 | newly | 3231. | 24 164.4 | {2652., 64 311.} | 656.768 | 0.707893 | Reff | 2.17415 | 0.925331 | {0.440051, 2.07453} | 57.4954 | ar | 98 881. | 146 145. | {78 220., 223 702.} | 55.1596 |
| 6 | R0 | {1.57191, 6.89004} | {2.3153, 4.89149} | {{1.75535, 1.18098}, {3., 12.5582}} | {47.292, 62.9315} | inf | 1343. | 27 926.3 | {4702.43, 61 207.9} | 1327.59 | 0.992474 | newly | 3690. | 29 104. | {4808., 74 471.} | 698.923 | 0.761751 | Reff | 1.49482 | 0.552699 | {0.177758, 0.9694} | 64.3393 | ar | 95 046. | 171 160. | {116 835., 262 758.} | 85.1679 |
| 7 | R0 | {1.99226, 17.2339} | {2.29901, 5.60362} | {{0.913065, 0.862902}, {3., 18.3566}} | {43.5404, 70.3535} | inf | 7484. | 5909.72 | {1161.8, 6715.2} | 66.2272 | 0.578843 | newly | 6848. | 7507.26 | {680., 10 576.} | 75.2085 | 0.450111 | Reff | 1.39466 | 0.519455 | {0.103362, 0.962806} | 75.4347 | ar | 97 992. | 188 791. | {170 072., 258 668.} | 92.6599 |
| 8 | R0 | {2.63369, 15.1879} | {2.0675, 4.30716} | {{1.03258, 2.65614}, {3., 4.94691}} | {33.762, 71.6409} | inf | 3695. | 11 982.1 | {3728.91, 21 688.6} | 117.246 | 0.311589 | newly | 4729. | 12 395.7 | {3566., 32 918.} | 171.473 | 0.341386 | Reff | 2.31741 | 0.366878 | {0.0408497, 0.777379} | 84.1686 | ar | 99 451. | 158 767. | {87 182., 194 494.} | 65.1785 |
| 9 | R0 | {2.53271, 9.90552} | {2.43261, 6.82899} | {{2.16411, 4.34816}, {3., 12.0111}} | {17.7926, 42.2008} | inf | 823. | 21 450.2 | {4133.67, 39 092.6} | 1605.39 | 1.10865 | newly | 2227. | 24 561. | {4503., 72 250.} | 1010.86 | 0.901428 | Reff | 2.46668 | 1.02138 | {0.269461, 2.18671} | 58.5929 | ar | 99 355. | 160 301. | {94 562., 240 797.} | 67.4154 |
| 10 | R0 | {2.48791, 18.9003} | {2.63195, 4.94823} | {{1.99529, 1.50279}, {3., 7.2519}} | {19.7334, 73.8193} | inf | 5017. | 1638.8 | {815.508, 1934.67} | 84.6465 | 0.867706 | newly | 4646. | 2578.96 | {759., 3698.} | 87.8769 | 0.620749 | Reff | 2.05964 | 0.518339 | {0.128622, 1.03658} | 74.8335 | ar | 99 291. | 178 087. | {166 966., 201 784.} | 85.06 |
| 11 | R0 | {2.84414, 18.4765} | {2.40879, 6.6034} | {{1.45131, 3.32498}, {3., 10.1674}} | {20.787, 64.2605} | inf | 3971. | 3337.73 | {1077.49, 9818.43} | 80.6244 | 0.565097 | newly | 4035. | 5257.93 | {618., 27 962.} | 139.872 | 0.550723 | Reff | 2.47881 | 0.599718 | {0.0811275, 1.4513} | 75.8263 | ar | 99 654. | 186 080. | {170 243., 249 893.} | 86.7259 |
| 12 | R0 | {2.29369, 14.622} | {2.57207, 4.68043} | {{1.99186, 1.03912}, {3., 7.51735}} | {23.5838, 67.9905} | inf | 4819. | 1476.01 | {267.892, 2105.87} | 83.6936 | 0.913519 | newly | 5911. | 3270.38 | {517., 4097.} | 101.359 | 0.786625 | Reff | 1.91186 | 0.541157 | {0.15951, 1.6776} | 72.3313 | ar | 98 982. | 180 608. | {166 391., 229 514.} | 84.0981 |
| 13 | R0 | {2.73413, 16.7529} | {2.04911, 3.9928} | {{0.0407624, 0.923929}, {3., 5.69943}} | {34.973, 76.1665} | inf | 4263. | 5842.36 | {463.834, 18 478.4} | 77.7687 | 0.480773 | newly | 4789. | 6395.52 | {417., 17 867.} | 94.474 | 0.434842 | Reff | 2.35852 | 0.336055 | {0.00515864, 0.432372} | 85.7514 | ar | 99 543. | 165 924. | {141 598., 234 384.} | 74.83 |
| 14 | R0 | {1.79554, 16.5943} | {2.73827, 4.12931} | {{2.19219, 1.90345}, {3., 6.16274}} | {52.5037, 75.1161} | inf | 6015. | 10 089.7 | {998.24, 9042.76} | 121.533 | 0.604877 | newly | 5421. | 16 860.5 | {1748., 15 930.} | 286.233 | 0.44779 | Reff | 1.3756 | 0.633289 | {0.185728, 2.09524} | 71.6909 | ar | 96 821. | 198 047. | {171 631., 283 790.} | 104.549 |
| 15 | R0 | {1.91429, 13.7771} | {2.38376, 6.29264} | {{0.927842, 0.6452}, {3., 19.5413}} | {51.3037, 75.2438} | inf | 5411. | 5594.68 | {67.0083, 5772.79} | 61.531 | 0.57457 | newly | 6031. | 7975.47 | {70., 9821.} | 102.199 | 0.644836 | Reff | 1.53354 | 0.37435 | {0.111695, 0.530807} | 77.5731 | ar | 97 541. | 184 912. | {167 349., 231 431.} | 89.574 |
| 16 | R0 | {1.42094, 10.5871} | {2.18032, 7.58655} | {{0.334054, 2.85264}, {3., 12.8608}} | {90.1137, 42.8387} | inf | 3733. | 3113.65 | {199.537, 2809.33} | 87.1915 | 0.742434 | newly | 4920. | 2847.55 | {134., 7761.} | 93.903 | 0.814401 | Reff | 1.20628 | 0.414674 | {0.0632668, 0.647398} | 70.3344 | ar | 93 044. | 185 059. | {175 362., 207 197.} | 98.8944 |
| 17 | R0 | {1.45515, 6.16466} | {2.53862, 6.65509} | {{2.1667, 4.16435}, {3., 14.6669}} | {78.6369, 45.1049} | inf | 733. | 19 525.8 | {8155.37, 32 505.4} | 1900.59 | 1.21628 | newly | 2188. | 24 603.7 | {5980., 72 028.} | 1024.73 | 0.883514 | Reff | 1.41733 | 0.967249 | {0.308283, 2.09724} | 51.1624 | ar | 93 254. | 151 470. | {86 101., 232 967.} | 66.6919 |
| 18 | R0 | {2.85669, 13.2416} | {2.32487, 4.82919} | {{1.76183, 1.12337}, {3., 12.8035}} | {22.2826, 63.5302} | inf | 1540. | 27 088.7 | {4618.35, 55 966.8} | 999.012 | 0.895994 | newly | 2807. | 31 936.4 | {4648., 68 728.} | 1042.94 | 0.903281 | Reff | 2.71777 | 0.637436 | {0.253334, 1.37195} | 76.5456 | ar | 99 644. | 163 508. | {66 114., 253 124.} | 75.975 |
| 19 | R0 | {2.56417, 17.9558} | {2.06202, 8.49614} | {{0.40235, 2.76848}, {3., 13.2123}} | {36.5801, 52.683} | inf | 4153. | 6284.51 | {2630.67, 29 683.9} | 103.109 | 0.464169 | newly | 4054. | 4601.71 | {1125., 17 755.} | 106.128 | 0.432264 | Reff | 2.21442 | 0.37551 | {0.0257909, 0.634749} | 83.0425 | ar | 99 389. | 184 645. | {173 725., 243 582.} | 85.7803 |
| 20 | R0 | {2.27517, 11.1864} | {2.36222, 4.54645} | {{1.76724, 1.33511}, {3., 12.5152}} | {24.7006, 64.7254} | inf | 2051. | 24 030.9 | {4318.84, 60 168.2} | 684.053 | 0.755029 | newly | 3692. | 28 768. | {4390., 72 135.} | 684.284 | 0.731499 | Reff | 2.12451 | 0.58521 | {0.14168, 1.27226} | 72.4953 | ar | 98 894. | 146 166. | {91 023., 235 197.} | 58.1405 |
| 21 | R0 | {2.35735, 10.4735} | {2.54841, 6.14075} | {{2.16923, 4.22807}, {3., 9.77708}} | {18.4374, 45.4461} | inf | 1087. | 21 758.5 | {6628.97, 36 068.5} | 1250.86 | 1.03429 | newly | 2417. | 28 617.1 | {6432., 69 908.} | 1086.33 | 0.910007 | Reff | 2.2751 | 1.00396 | {0.216509, 2.18181} | 56.1057 | ar | 99 036. | 156 516. | {89 855., 228 216.} | 64.7198 |
| 22 | R0 | {1.79551, 11.9179} | {2.43313, 4.73022} | {{1.12879, 1.42413}, {3., 7.19081}} | {43.7886, 60.3099} | inf | 4439. | 2042.13 | {192.525, 2515.8} | 87.6857 | 0.84771 | newly | 5958. | 2986.72 | {225., 4869.} | 89.2722 | 0.738514 | Reff | 1.5037 | 0.558621 | {0.123846, 1.85776} | 70.0781 | ar | 97 033. | 189 018. | {174 571., 249 677.} | 95.0499 |
| 23 | R0 | {1.23545, 6.31687} | {2.04134, 3.92141} | {{0.0425342, 0.943987}, {3., 5.75356}} | {109.616, 43.3746} | inf | 2814. | 5548.64 | {1159.42, 16 302.1} | 76.0352 | 0.260908 | newly | 6598. | 6085.34 | {1011., 16 177.} | 67.442 | 0.379109 | Reff | 1.0892 | 0.338925 | {0.00577306, 0.455751} | 74.9259 | ar | 88 739. | 168 864. | {150 993., 227 120.} | 96.478 |
| 24 | R0 | {2.24089, 10.1789} | {2.45934, 4.84412} | {{1.76005, 1.12095}, {3., 12.8035}} | {27.0577, 62.1181} | inf | 976. | 18 675.1 | {4373.48, 50 136.2} | 1239.05 | 0.961898 | newly | 2027. | 21 539.3 | {2515., 44 336.} | 964.581 | 0.827818 | Reff | 2.17337 | 0.572436 | {0.166466, 1.05477} | 73.6614 | ar | 98 868. | 148 424. | {89 219., 248 190.} | 56.3477 |
| 25 | R0 | {1.57298, 10.2607} | {2.40545, 7.32091} | {{1.41172, 3.35389}, {3., 9.81934}} | {58.0398, 28.651} | inf | 2641. | 2779.94 | {88.7303, 1526.96} | 96.6368 | 0.597702 | newly | 3941. | 3844.57 | {50., 2204.} | 131.838 | 0.731608 | Reff | 1.41523 | 0.474476 | {0.077256, 0.847569} | 70.2553 | ar | 94 957. | 180 374. | {167 579., 183 575.} | 89.9535 |
| 26 | R0 | {1.2154, 8.2576} | {2.4949, 11.9904} | {{1.27749, 4.73073}, {3., 17.3245}} | {105.274, 65.6975} | inf | 4010. | 4272.46 | {1356.55, 12 660.7} | 86.8122 | 0.49335 | newly | 6408. | 5653.41 | {541., 7530.} | 133.119 | 0.786363 | Reff | 0.993966 | 0.684735 | {0.169419, 2.09428} | 67.5875 | ar | 88 421. | 193 325. | {171 650., 277 409.} | 118.642 |
| 27 | R0 | {2.22841, 18.7983} | {2.49457, 12.0817} | {{1.22475, 4.75352}, {3., 17.4563}} | {29.0833, 35.7299} | inf | 7582. | 3887.1 | {639.826, 5953.03} | 84.9423 | 0.887837 | newly | 6511. | 5146.06 | {465., 8439.} | 125.478 | 0.889171 | Reff | 1.58429 | 0.744959 | {0.262607, 2.10453} | 62.9261 | ar | 98 756. | 190 174. | {168 868., 253 383.} | 92.5694 |
| 28 | R0 | {1.27778, 5.36767} | {2.51006, 7.21057} | {{2.17818, 4.1003}, {3., 11.0494}} | {98.3478, 51.2041} | inf | 966. | 24 373.5 | {1147.11, 55 941.2} | 1854.83 | 1.11941 | newly | 2971. | 24 312. | {1623., 59 178.} | 732.793 | 0.791372 | Reff | 1.23118 | 0.983933 | {0.410978, 2.18213} | 50.6227 | ar | 89 843. | 151 334. | {85 960., 219 008.} | 73.6519 |
| 29 | R0 | {2.38042, 15.8901} | {2.10352, 4.42853} | {{0.0674053, 0.912423}, {3., 5.83394}} | {40.7852, 72.1302} | inf | 3337. | 4235.22 | {705.819, 6529.25} | 72.6571 | 0.427942 | newly | 3642. | 4600.61 | {616., 13 814.} | 96.6395 | 0.419105 | Reff | 2.11469 | 0.330928 | {0.00961732, 0.458699} | 84.351 | ar | 99 112. | 156 514. | {86 027., 182 686.} | 66.0989 |
| 30 | R0 | {2.53188, 12.7607} | {2.50296, 6.81565} | {{2.16002, 4.17947}, {3., 14.7215}} | {17.4125, 49.985} | inf | 1016. | 20 102.6 | {2195., 34 395.5} | 1328.51 | 1.015 | newly | 1649. | 22 529.3 | {2975., 59 303.} | 1270.55 | 0.947002 | Reff | 2.45506 | 0.900214 | {0.307771, 2.18587} | 63.3323 | ar | 99 317. | 140 839. | {66 937., 216 182.} | 56.3243 |
| 31 | R0 | {1.24482, 10.5471} | {2.71551, 4.56697} | {{2.1466, 1.77521}, {3., 6.19805}} | {118.145, 56.6993} | inf | 5483. | 4921.31 | {1412.75, 7824.93} | 84.6552 | 0.534663 | newly | 6713. | 8482.09 | {1839., 7893.} | 138.049 | 0.481336 | Reff | 0.914831 | 0.511296 | {0.194039, 0.751149} | 56.5971 | ar | 89 097. | 181 024. | {165 360., 218 109.} | 103.176 |
| 32 | R0 | {2.30446, 17.9607} | {2.52219, 4.88387} | {{1.98595, 1.4241}, {3., 7.23054}} | {23.7526, 72.808} | inf | 5816. | 2177.99 | {473.915, 2781.} | 85.0872 | 0.891507 | newly | 5244. | 2482.65 | {646., 4402.} | 77.512 | 0.637017 | Reff | 1.82382 | 0.478274 | {0.174911, 0.655486} | 74.9623 | ar | 99 028. | 159 721. | {149 285., 185 618.} | 66.6138 |
| 33 | R0 | {2.31579, 11.8236} | {2.39648, 6.44406} | {{2.03412, 4.10625}, {3., 9.68412}} | {18.8633, 48.9955} | inf | 3092. | 23 561.4 | {6772.67, 48 744.} | 406.91 | 0.610269 | newly | 5187. | 27 301. | {6087., 67 526.} | 433.846 | 0.598852 | Reff | 2.08296 | 1.00466 | {0.280892, 2.18692} | 53.7276 | ar | 99 012. | 166 818. | {85 190., 241 938.} | 74.6533 |
| 34 | R0 | {1.6678, 10.6744} | {2.43462, 4.4234} | {{0.980622, 1.39188}, {3., 7.2038}} | {54.2907, 58.5607} | inf | 4713. | 1998.19 | {980.766, 3699.31} | 79.4985 | 0.737763 | newly | 6918. | 3600.53 | {634., 12 734.} | 89.9394 | 0.717811 | Reff | 1.37288 | 0.531708 | {0.131078, 1.13014} | 67.9862 | ar | 95 809. | 182 316. | {169 976., 211 277.} | 91.9455 |
| 35 | R0 | {1.65476, 7.07559} | {2.60243, 7.29925} | {{2.16851, 4.28724}, {3., 14.7892}} | {59.0697, 44.9001} | inf | 893. | 22 231. | {5823.42, 37 625.} | 1673.63 | 1.12103 | newly | 2514. | 23 890.8 | {5761., 71 771.} | 856.65 | 0.822507 | Reff | 1.60409 | 1.00157 | {0.345972, 2.12114} | 52.3871 | ar | 95 746. | 162 264. | {93 578., 247 881.} | 75.5871 |
| 36 | R0 | {2.71807, 18.5959} | {2.42896, 5.08038} | {{1.29878, 1.54759}, {3., 7.29576}} | {20.8014, 72.6801} | inf | 3397. | 2130.21 | {779.258, 2621.34} | 84.0893 | 0.640544 | newly | 3163. | 2409.51 | {732., 4569.} | 82.0711 | 0.462562 | Reff | 2.4286 | 0.507281 | {0.154206, 1.2892} | 79.1122 | ar | 99 540. | 185 607. | {169 839., 209 897.} | 86.4645 |
| 37 | R0 | {2.6202, 12.7552} | {2.16807, 3.88362} | {{0.0465646, 0.931815}, {3., 5.71946}} | {34.7703, 69.8778} | inf | 1248. | 5858.44 | {536.219, 19 709.8} | 251.357 | 0.47374 | newly | 2178. | 7896.68 | {504., 17 943.} | 303.271 | 0.508612 | Reff | 2.51914 | 0.369752 | {0.00627019, 0.598239} | 85.3223 | ar | 99 426. | 165 968. | {97 981., 234 119.} | 73.454 |
| 38 | R0 | {1.47672, 9.81651} | {2.1168, 9.05982} | {{0.339704, 2.66588}, {3., 13.559}} | {84.8287, 42.16} | inf | 4210. | 5726.47 | {89.3665, 29 232.3} | 114.939 | 0.86601 | newly | 6266. | 4418.2 | {104., 21 215.} | 105.598 | 0.987127 | Reff | 1.23 | 0.4476 | {0.063599, 0.639281} | 66.931 | ar | 93 815. | 187 666. | {170 475., 241 563.} | 100.039 |
| 39 | R0 | {1.90004, 12.3313} | {2.53494, 4.77564} | {{2.00539, 1.45686}, {3., 7.28496}} | {37.6914, 61.2722} | inf | 3615. | 1604.83 | {786.591, 2896.73} | 78.3457 | 0.690829 | newly | 4980. | 2303.26 | {677., 4358.} | 79.2606 | 0.637797 | Reff | 1.65284 | 0.527816 | {0.165069, 1.51923} | 71.8465 | ar | 97 413. | 178 302. | {159 987., 234 968.} | 83.5561 |
| 40 | R0 | {2.64218, 18.5237} | {2.1067, 4.20064} | {{0.00259498, 0.921299}, {3., 5.68628}} | {34.6483, 77.3229} | inf | 6412. | 4907.13 | {1098.96, 10 070.9} | 69.2287 | 0.568525 | newly | 6088. | 5195.69 | {814., 11 454.} | 65.1979 | 0.408328 | Reff | 2.0572 | 0.284792 | {0.000528369, 0.461543} | 86.1563 | ar | 99 474. | 156 851. | {141 568., 185 281.} | 63.973 |
| 41 | R0 | {1.6945, 7.17996} | {2.61518, 7.13699} | {{2.1627, 4.17745}, {3., 14.7305}} | {54.9628, 44.1234} | inf | 498. | 22 593.7 | {7151.68, 34 848.4} | 3365.29 | 1.42564 | newly | 1333. | 25 510.6 | {7056., 74 370.} | 1814.24 | 1.1104 | Reff | 1.66739 | 0.961458 | {0.439256, 2.14507} | 52.3749 | ar | 96 332. | 149 158. | {78 282., 233 923.} | 62.7475 |
| 42 | R0 | {1.84559, 18.0074} | {2.4455, 5.6746} | {{0.91552, 0.842844}, {3., 18.4087}} | {54.3165, 69.5053} | inf | 7319. | 5664.18 | {1252.5, 5794.42} | 67.7297 | 0.556374 | newly | 6151. | 9672.47 | {1137., 12 284.} | 119.429 | 0.459985 | Reff | 1.29117 | 0.561455 | {0.101684, 0.689153} | 62.7109 | ar | 97 358. | 196 374. | {178 092., 238 590.} | 101.703 |
| 43 | R0 | {2.24965, 17.0361} | {2.59345, 5.17515} | {{1.99981, 1.49661}, {3., 7.23411}} | {25.8523, 69.6224} | inf | 4414. | 2161.61 | {747.592, 2534.27} | 84.4014 | 0.733531 | newly | 4281. | 2711.67 | {829., 5011.} | 91.4083 | 0.564327 | Reff | 1.90728 | 0.481543 | {0.19568, 1.0158} | 75.1403 | ar | 98 770. | 175 340. | {163 475., 202 039.} | 79.1889 |
| 44 | R0 | {2.54324, 15.7967} | {2.48117, 4.75448} | {{1.98839, 1.4606}, {3., 7.32251}} | {20.0411, 69.9021} | inf | 4842. | 2192.21 | {542.541, 2751.58} | 82.7862 | 0.768983 | newly | 5449. | 3323.64 | {765., 4912.} | 82.4382 | 0.600937 | Reff | 2.13836 | 0.588808 | {0.151931, 1.95449} | 72.4645 | ar | 99 354. | 190 867. | {171 930., 253 246.} | 92.1084 |
| 45 | R0 | {2.54943, 14.8032} | {2.13065, 4.11571} | {{1.07813, 2.65362}, {3., 4.93982}} | {33.045, 72.1972} | inf | 3565. | 14 414.3 | {4429.41, 30 321.} | 167.372 | 0.354689 | newly | 4552. | 16 329.4 | {5577., 44 261.} | 267.817 | 0.446385 | Reff | 2.25387 | 0.355969 | {0.0727716, 0.805355} | 84.2064 | ar | 99 380. | 159 915. | {85 136., 200 058.} | 68.8035 |
| 46 | R0 | {2.37755, 12.6461} | {2.52842, 6.97817} | {{2.17303, 4.13826}, {3., 14.7514}} | {18.7873, 49.4325} | inf | 1629. | 23 160.4 | {5243.2, 52 806.9} | 798.201 | 0.830168 | newly | 2717. | 25 520.6 | {6150., 73 331.} | 841.635 | 0.806245 | Reff | 2.25026 | 0.987903 | {0.448072, 2.15164} | 56.1937 | ar | 99 096. | 148 183. | {72 984., 232 819.} | 61.1623 |
| 47 | R0 | {1.69279, 11.6636} | {2.42305, 6.35628} | {{1.43396, 3.33008}, {3., 9.79983}} | {50.777, 45.5033} | inf | 3683. | 3054.3 | {1338.58, 2600.9} | 91.705 | 0.65737 | newly | 4864. | 3875.1 | {710., 5788.} | 112.551 | 0.68724 | Reff | 1.4668 | 0.54193 | {0.074831, 0.970774} | 64.471 | ar | 96 214. | 189 740. | {177 440., 203 042.} | 97.206 |
| 48 | R0 | {1.82402, 10.0038} | {2.19383, 4.5259} | {{1.15215, 2.64635}, {3., 5.0055}} | {48.9464, 54.7582} | inf | 1751. | 13 194.3 | {4909.11, 24 464.7} | 412.712 | 0.588529 | newly | 3183. | 14 365.2 | {3231., 43 713.} | 356.149 | 0.492836 | Reff | 1.71321 | 0.371093 | {0.0747989, 0.733778} | 79.4187 | ar | 97 076. | 158 204. | {90 992., 217 739.} | 68.705 |
| 49 | R0 | {1.6424, 11.1667} | {2.51053, 4.62018} | {{1.9905, 1.36599}, {3., 7.17186}} | {56.3172, 58.6254} | inf | 5534. | 1825.63 | {784.928, 2566.29} | 85.3618 | 0.824133 | newly | 7611. | 2461.89 | {809., 4771.} | 79.9418 | 0.733813 | Reff | 1.28365 | 0.497941 | {0.140096, 1.06917} | 69.8407 | ar | 95 546. | 178 291. | {165 005., 197 762.} | 86.6021 |
| 50 | R0 | {2.05638, 14.3048} | {1.84906, 3.94478} | {{0.0768619, 0.890658}, {3., 5.71988}} | {53.1145, 72.5242} | inf | 4454. | 6918.4 | {825.469, 19 950.8} | 89.615 | 0.469319 | newly | 5024. | 8433.89 | {777., 18 368.} | 135.036 | 0.445953 | Reff | 1.73898 | 0.301774 | {0.0101195, 0.460692} | 82.6465 | ar | 98 194. | 173 262. | {156 075., 234 060.} | 81.6838 |
